# Effect of Thermal Shock on Properties of a Strongly Amorphous AlCrTiZrMo High-Entropy Alloy Film

**DOI:** 10.3390/ma16165629

**Published:** 2023-08-15

**Authors:** Shunian Chen, Weiqing Yan, Yifan Zhang, Lin Chen, Xiaoping Ouyang, Xiao Ouyang, Jing Chen, Bin Liao

**Affiliations:** 1 Key Laboratory of Beam Technology of Ministry of Education, College of Nuclear Science and Technology, Beijing Normal University, Beijing 100875, China; 18811573685@163.com (S.C.); 17862722263@163.com (W.Y.); 202131220014@mail.bnu.edu.cn (Y.Z.); oyxp2003@aliyun.com (X.O.); oyx16@tsinghua.org.cn (X.O.); 2 Advanced Institute of Natural Sciences, Beijing Normal University at Zhuhai, Zhuhai 519087, China; lchen1209@163.com; 3 School of Materials Science and Engineering, Xiangtan University, Xiangtan 411105, China

**Keywords:** AlCrTiZrMo high-entropy alloy film, thermal shock, oxidation, tribocorrosion

## Abstract

AlCrTiZrMo high-entropy alloy (HEA) films with strong amorphization were obtained by co-filter cathode vacuum arc deposition, and the effect of thermal shock on the films was investigated in order to explore the protection mechanism of HEA films against mechanical components in extreme service environments. The results show that after annealing at 800 °C for 1 h, the formation of a dense ZrTiO_4_ composite oxide layer on the surface actively prevents the oxidation from continuing, so that the AlCrTiZrMo HEA film exhibits excellent oxidation resistance at 800 °C in air. In the friction-corrosion coupling environment, the AlCrTiZrMo HEA film annealed at 800 °C for 1 h shows the best tribocorrosion resistance due to the stable dense microstructure and excellent mechanical properties, and its ΔOCP, COF and wear rate possess the smallest values of 0.055, 0.04 and 1.34 × 10^−6^ mm^−3^·N^−1^·m^−1^.

## 1. Introduction

In marine engineering, the materials of mechanical components need to have service reliability in extremely harsh working environments, that is, on the basis of excellent high-temperature mechanical properties, they must also have the ability to resist the erosion of oxygen or corrosive media in high-temperature environments. The oxidation, friction and corrosion of the materials of power components are the process of chemical or electrochemical reactions between the materials and the medium in the surrounding environment, which leads to the failure of the materials. Therefore, the development of protective coatings and the study of the effect of thermal shock on the performance of protective coatings are particularly important.

Since the study of high-entropy alloy (HEA), its excellent properties have attracted extensive attention [1]. HEA film, as a low-dimensional material of HEA, has a more uniform element distribution and is superior to bulk HEA in many properties [2,3]. According to the research, through the rational design of an alloy system, HEA films can exhibit many excellent properties in terms of wear resistance [4,5], corrosion resistance [6] and high temperature oxidation [7]. Therefore, as a breakthrough in the field of traditional alloys, HEA films have broad application prospects [8,9]. The development of HEAs is rapid, but it is still in the stage of basic research. The research on HEA films mainly focuses on the preparation of HEA nitride, oxide and carbide films and the influence of process parameters on the film properties. The mechanism research, functional design and process optimization of HEA films still need to be further explored. However, the experimental preparation of HEA films has been a challenge. Magnetron sputtering provides an opportunity for the research of HEA films [10,11,12]. But the high cost, low ionization rate and uncontrollable composition caused by the preparation technology are the problems restricting the optimization of HEA thin films. Therefore, the improvement of the preparation process is crucial for the development of HEA films.

Filter cathode vacuum arc deposition (FCVAD) has sufficient advantages to obtain multi-component films due to its characteristics of magnetic filtration, high ionization rate (100%) and controllable ion types and ion energy. Co-filter cathode vacuum arc deposition (Co-FCVAD) is obtained by designing a multi-channel for particle transport based on FCVAD, which significantly improves the defects of uncontrollable composition and poor quality in the preparation of HEA films [13,14].

Thus, in order to improve the performance of mechanical components in extreme service environments, the AlCrTiZrMo film system is determined by selecting elements with a large atomic radius difference and excellent corrosion and high temperature performance. In this work, the AlCrTiZrMo HEA film is prepared based on Co-FCVAD. The synergistic interaction between elements at a high temperature in air is discussed, and the changes in microstructure, mechanical properties and tribocorrosion properties of the film after annealing at different temperatures are studied, so as to analyze the protection mechanisms of AlCrTiZrMo HEA films in the simulated scenario of thermal shock.

## 2. Experimental Details

**Figure 1:**
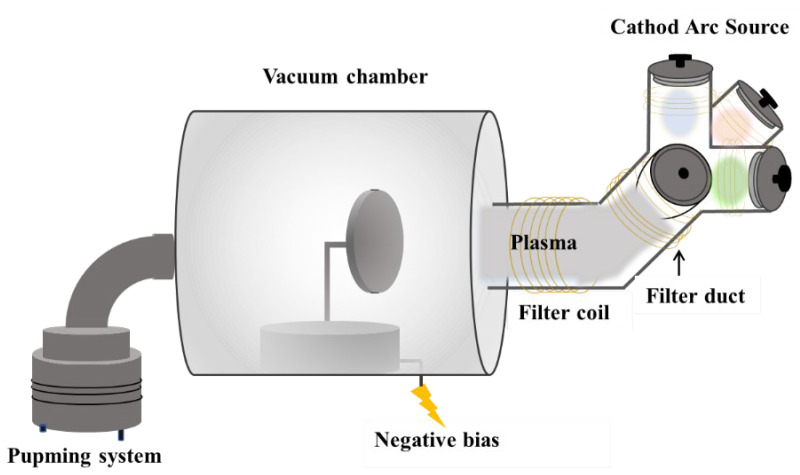
Schematic diagram of the Co-FCVAD system.

AlCrTiZrMo HEA film was prepared on 304 stainless steel (20 × 20 × 2 mm) and silicon wafer (20 × 20 × 0.5 mm) by Co-FCVAD (Figure 1). Four metal targets of 99.99% purity Ti50Mo50, pure Al, Cr and Zr were ionized at the same time for co-deposition of AlCrTiZrMo HEA films. Surface stains were removed by washing the substrate successively in acetone and ethanol for 5 min. Prior to deposition, the substrate was sputtered sequentially for 45 s at negative bias voltages of −800, −600 and −400 V. Detailed process parameters are shown in Table 1.

In this work, the microstructural characterization of the films was evaluated using a scanning electron microscope (SEM) of Hitachi S-4800, an energy dispersive spectrometer (EDS) of EMAX-350 and a high-resolution transmission electron microscope (HRTEM) of FEI-Tecnai G20. The phase structure of the film was detected by X-ray diffraction (XRD, SmartLab S2). The hardness and Young’s modulus of the film were measured using a Nanoindenter G200 (Keysight Technologies). High temperature oxidation in an air environment was carried out through a tube furnace with a heating rate of 10 °C/min during the test.

The tribocorrosion mechanism of the film was analyzed by a linear reciprocating friction machine configured with three electrodes. Si_3_N_4_ ceramic grinding balls (Φ 6 mm) were slid for 30 min at a load of 5 N and a frequency of 1 Hz. Prior to formal testing, the sample was immersed in the electrolytic cell with the corrosion solution until the surface potential was stable. The changes in open circuit potential (OCP) and coefficient of friction (COF) were collected in real time throughout the experiment. The wear rate was obtained by the formula *w* = *V*/*F* × *S*. And the meanings of each physical quantity are as follows: *V* (mm^3^) represents wear volume, *F* (N) represents applied load and *S* (m) represents sliding distance.

## 3. Results and Discussion

### 3.1. Composition and Structural Characteristics

**Figure 2:**
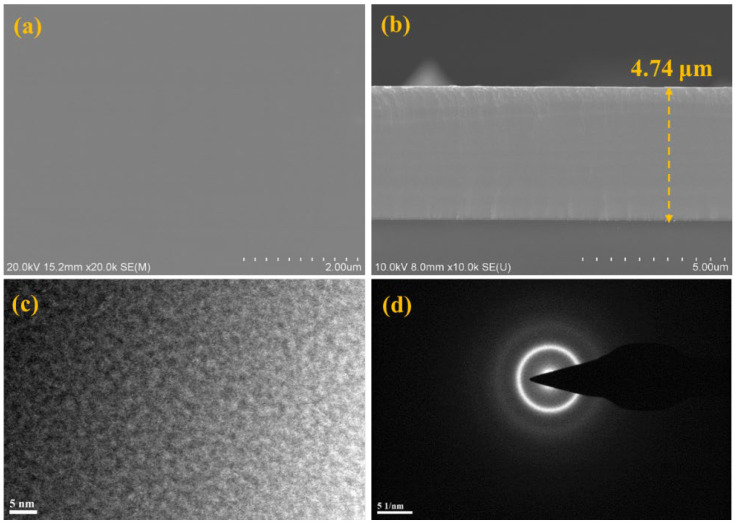
(**a**) SEM surface morphology, (**b**) SEM cross-section morphology, (**c**) HRTEM image and (**d**) the corresponding selected area electron diffraction (SAED) pattern of AlCrTiZrMo HEA film.

**Figure 3:**
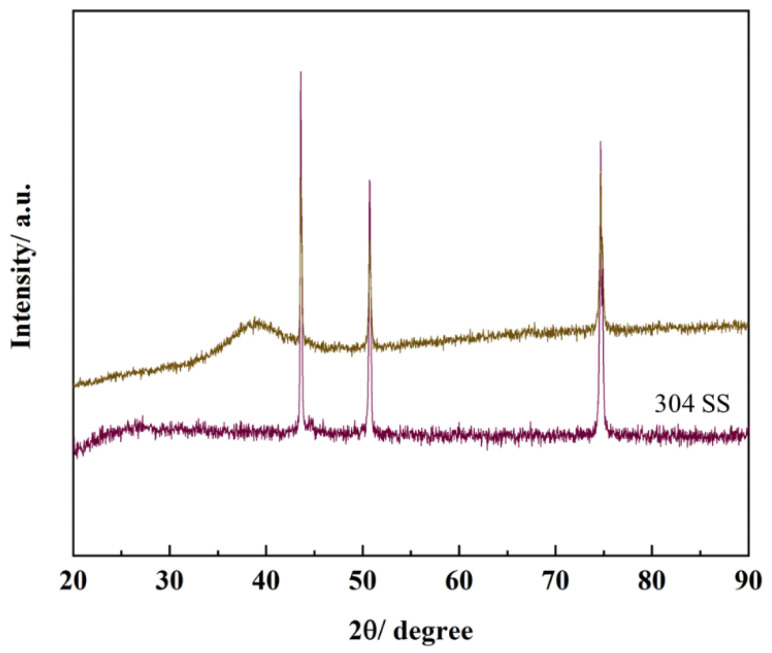
XRD pattern of AlCrTiZrMo HEA film.

As shown in Table 2, the relative contents of Al, Cr, Ti, Zr and Mo in AlCrTiZrMo films are 15.82 at.%, 22.86 at.%, 19.49 at.%, 24.74 at.% and 17.09 at.%, respectively, which do not deviate from the 5–35 at.% element rule defined by HEA. It can be observed from Figure 2a,b that AlCrTiZrMo HEA film is uniform and dense. From the XRD pattern shown in Figure 3, except for the diffraction peak of the substrate, only the broad diffraction peak with amorphous characteristics appears. In addition, the results of TEM analysis show that the AlCrTiZrMo HEA film exhibits strong amorphization, the disorder microstructure exists in the film and the diffraction rings are weaker and broader (Figure 2c,d).

Many researchers have concluded through a large number of experiments that the amorphous phase is easy to form for HEA in the region of δ> 6.6%, −49 < $∆H_{mix}$ < −5.5 kJ/mol, 7 < $∆S_{conf}$ < 16 J/(K·mol), where δ is the atomic size polydispersity [15,16,17,18], Δ*H_mix_* is the mixing enthalpy [19,20] and Δ*S_conf_* is the configurational entropy [21].

**Figure 4:**
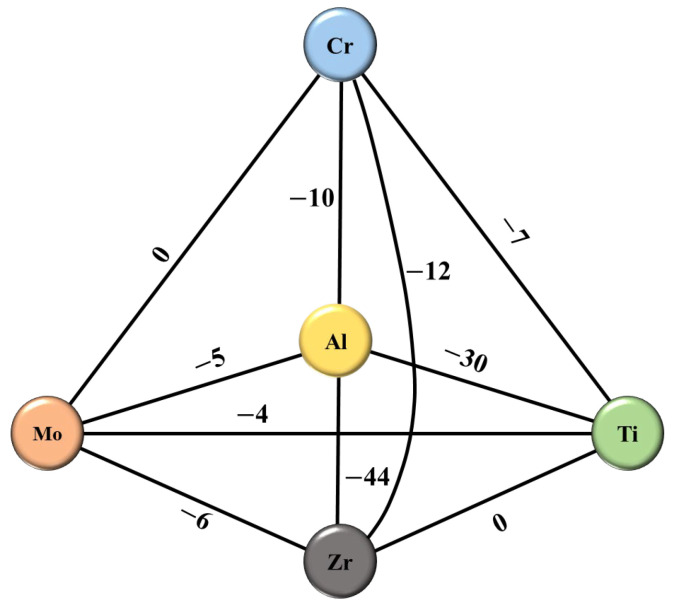
Mixing enthalpy (kJ/mol) of a binary alloy formed between the metal elements Al, Cr, Ti, Zr and Mo.

Δ=∑i=1nci1−rir¯2

where $\bar{r}=\sum_{i=1}^{n}c_{i}r_{i}$, $c_{i}$ and $r_{i}$ are the atomic percentage and atomic radius of the *i*th element, and *n* is the number of alloying elements.

$$∆H_{mix}=\sum_{i=1,i\neq j}^{n}c_{i}c_{j}\omega_{ij}$$

where $\omega_{ij}=4{∆}_{mix}^{CD}$; here, ${∆}_{mix}^{CD}$ denotes the enthalpy of the mixing of binary alloy CD.

$$∆S_{conf}=-R\sum_{i=1}^{n}c_{i}\ln c_{i}$$

where *R* is gas constant. The mixing enthalpy between binary alloys in AlCrTiZrMo is summarized by reference, as shown in Figure 4 [19]. The values of *δ*, Δ*H_mix_* and Δ*S_conf_* of the AlCrTiZrMo system are 0.077, −18.40 kJ/mol and 13.55 J/(K·mol), respectively, indicating that AlCrTiZrMo HEA films are easy to form amorphous structures. The above XRD and HRTEM results confirm the strong amorphization structural characteristics of the AlCrTiZrMo HEA film.

### 3.2. High-Temperature Annealing in Air Environment

**Figure 5:**
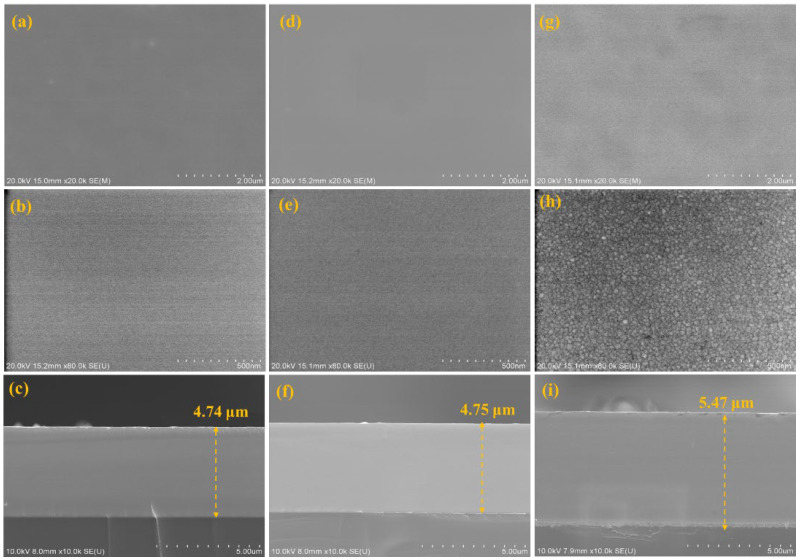
SEM surface and cross-section images of AlCrTiZrMo HEA film after annealing at 400 °C (**a**–**c**), 600 °C (**d**–**f**) and 800 °C (**g**–**i**) in an air environment.

**Figure 6:**
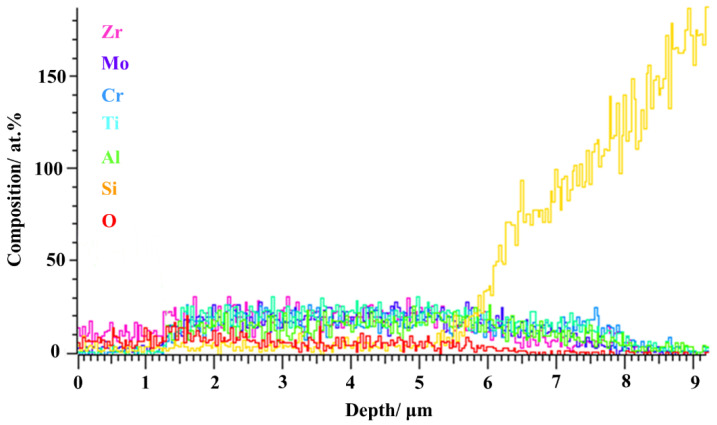
Cross-section EDS profiles of AlCrTiZrMo HEA film after annealing in air at 800 °C.

**Figure 7:**
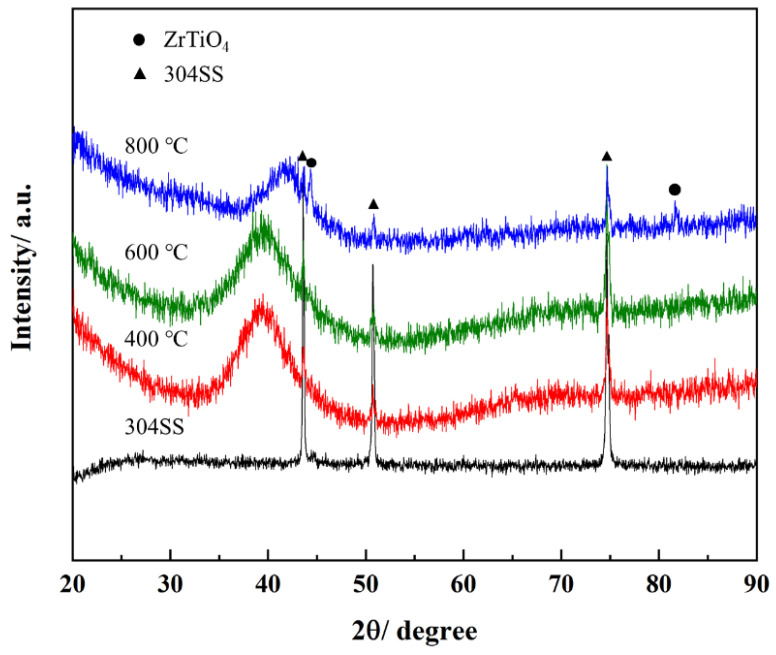
XRD patterns of AlCrTiZrMo HEA film after annealing in air at different temperatures of 400 °C, 600 °C and 800 °C.

The effect of thermal shock on AlCrTiZrMo HEA films can lead to differences in properties, so it is crucial to study the details of the high temperature oxidation of AlCrTiZrMo HEA films. The AlCrTiZrMo HEA films were annealed at 400 °C, 600 °C and 800 °C for 1 h in an air environment for analysis. It can be observed from Figure 5 that the AlCrTiZrMo HEA film still maintains a dense and uniform morphology after annealing at 400 °C and 600 °C, and no oxide particles appear. The thickness of the film does not change significantly. In addition, it can be seen from the XRD patterns that no crystallization peak appears in the respective pattern except for the diffraction peaks of the substrate. The slow diffusion effect of the AlCrTiZrMo HEA film and the amorphous phase in the structure make the diffusion of oxygen elements difficult, so the film does not demonstrate obvious oxidation after annealing at 600 °C, exhibiting excellent oxidation resistance. When the AlCrTiZrMo HEA film is annealed at 800 °C, it can be found in the enlarged surface morphology that the surface is covered by fine oxides (Figure 5h), and the thickness is increased to 5.47 μm (Figure 5i). At this time, the content of O element in the film increases to 20.50 at.% (Table 3). From the element distribution of the cross section in Figure 6 and the XRD pattern in Figure 7, it can be seen that the oxide is composed of the composite oxide ZrTiO_4_ [22,23,24,25].

As shown in Table 4, the oxidation thermodynamics of elemental Al, Cr, Ti, Zr and Mo indicate that the order of Gibbs free energy of oxide formation of each element at 800 °C is ZrO_2_ < Al_2_O_3_ < TiO_2_ < Cr_2_O_3_ < MoO_2_, illustrating the preferential oxidation of Zr [26]. Although the oxidation of Al is preferred to that of Ti in terms of oxidation thermodynamics, the difference in oxidation kinetics resulting from higher Ti content than Al leads to the preferential oxidation of Ti [27].

**Figure 8:**
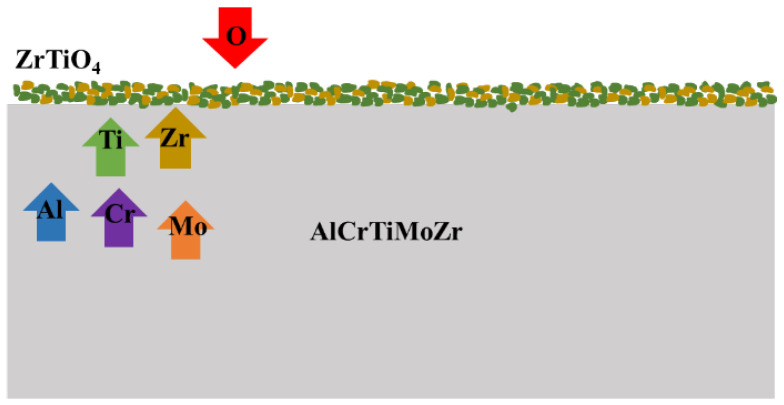
The schematic diagram of oxidation mechanism of AlCrTiZrMo HEA film after annealing at 800 °C in air.

Figure 8 shows the schematic diagram of the oxidation mechanism of the AlCrTiZrMo HEA film at 800 °C for 1 h. Zr and Ti elements are preferentially oxidized to form ZrO_2_ and TiO_2_. Meanwhile, metal elements that form low free energy oxides can undergo reduction reactions with oxides with high free energy. Thus, accompanied by the oxidation reaction, the TiO_2_ oxide will be reduced by the Zr element [28].

$$Zr+{TiO}_{2}=ZrO_{2}+Ti$$

The continuous competitive oxidation and reduction reactions between the Zr and Ti elements increase the accumulation of ZrO_2_ and TiO_2_ oxides, and the coarse oxides are gradually decomposed into fine oxide particles. With the process of high temperature oxidation, the ZrO_2_ and TiO_2_ oxides seek a decrease in free energy at the interface with each other and polymerize to form spinel ZrTiO_4_.

$$ZrO_{2}+{TiO}_{2}=Zr{TiO}_{4}$$

$$∆G_{Zr{TiO}_{4}}=∆G_{ZrO_{2}}+∆G_{TiO_{2}}=-893.255-751.310=-1644.565\;\mathrm{kJ/mol}$$

The value of the Gibbs free energy of formation for ZrTiO_4_ is very low, indicating that it is a stable substance. Therefore, ZrTiO_4_ is continuously generated and polymerized along the periphery of TiO_2_ particles, and finally forms a dense oxide layer on the surface. The XRD pattern in Figure 7 shows that the ZrTiO_4_ phase appears and no other oxides are found after annealing at 800 °C for 1 h, indicating that the dense ZrTiO_4_ composite oxide layer formed on the surface effectively prevents the oxidation from continuing, which means that the AlCrTiZrMo HEA film still shows excellent oxidation resistance at 800 °C for 1 h in an air environment.

### 3.3. Mechanical Properties after Annealing in Air Environment

**Figure 9:**
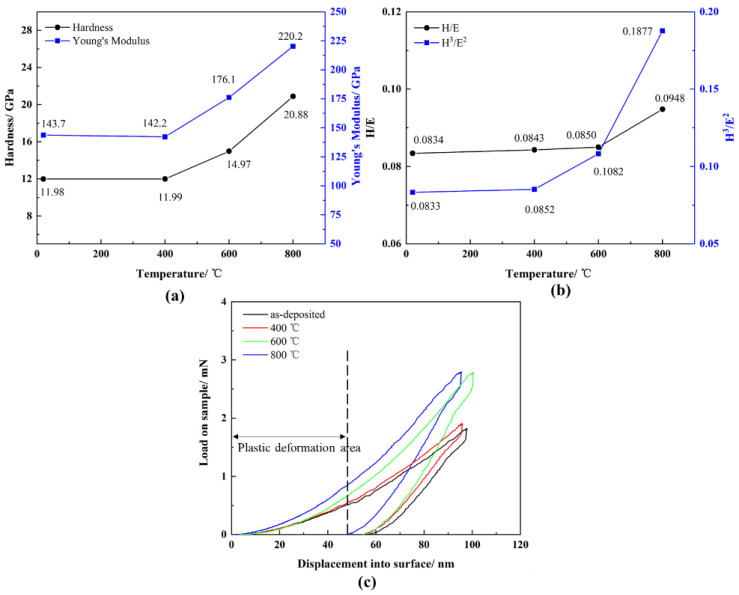
(**a**) H and E, (**b**) H/E and H^3^/E^2^ and (**c**) nanoindentation depth-load curves of AlCrTiZrMo HEA film after annealing in air at different temperatures of RT, 400 °C, 600 °C and 800 °C for 1 h.

After annealing at 400 °C for 1 h, there are no significant changes in the hardness (H) and elastic modulus (E), which are 11.99 GPa and 142.2 GPa, respectively (Figure 9). After annealing at 600 °C, due to the relaxation of the amorphous structure, the hardness and elastic modulus increase to 14.97 GPa and 176.1 GPa, respectively [29]. After annealing at 800 °C, the fine oxide particles precipitated have the effect of dispersion strengthening [30,31], resulting in the H and E of the film reaching 20.88 GPa and 220.2 GPa, respectively.

The load-displacement curves of the AlCrTiZrMo HEA film after annealing at different temperatures in air are described in Figure 9c. It can be found that the plastic deformation area of the film after annealing at 800 °C is narrower, indicating that it has excellent resistance to plastic deformation [32]. Moreover, it can be seen from Figure 9b that the H/E and H^3^/E^2^ of the AlCrTiZrMo HEA film annealed at 800 °C increase to their maximum values, which are 0.0948 and 0.1877, respectively. It is generally believed that the higher the H/E of the material, the better the crack resistance and wear resistance, and the higher the H^3^/E^2^, the better the resistance to plastic deformation [33,34].

### 3.4. Tribocorrosion Performance after Annealing in Air Environment

**Figure 10:**
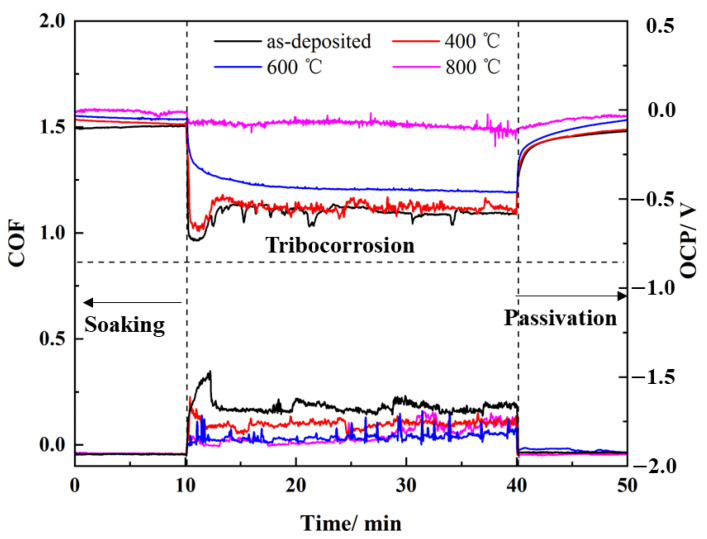
The COF and OCP curves of AlCrTiZrMo HEA film after annealing for 1 h in air at different temperatures of RT, 400 °C, 600 °C and 800 °C in 3.5wt.% NaCl solution.

The influence of thermal shock on the tribocorrosion properties of AlCrTiZrMo HEA films is analyzed by monitoring the fluctuation of the OCP and COF of films in the coupled environment of friction and corrosion, as shown in Figure 10. The fluctuation of the OCP and COF of the AlCrTiZrMo HEA film and the AlCrTiZrMo HEA film annealed at 400 °C, 600 °C and 800 °C for 1 h in air are monitored in real time. In the preparation stage, the samples were completely immersed in a 3.5 wt.% NaCl solution for 1 h to obtain a stable surface potential. The duration of the whole experiment is 50 min. During the no-load phase of 0–10 min, both the OCP and COF curves remain stable. During the 10–40 min loading stage, the appearance of the activation region causes the OCP curve to drop sharply, then fluctuate around a constant value. During the 40–50 min no-loading stage, the lack of load causes re-passivation within the wear track, and the OCP curve gradually returns to stability. Therefore, OCP can be used as a mixed potential to monitor the passivation state within the wear track [35,36,37].

**Figure 11:**
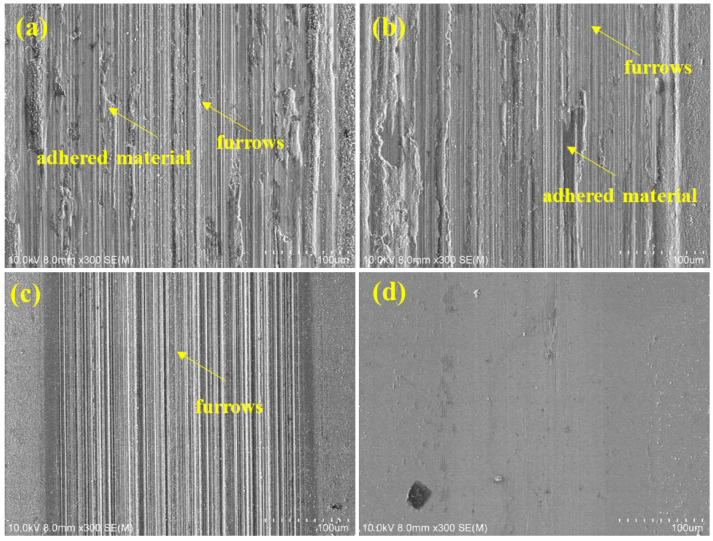
SEM morphology of wear tracks of AlCrTiZrMo HEA film after annealing in air at (**a**) RT, (**b**) 400 °C, (**c**) 600 °C and (**d**) 800 °C for 1 h in 3.5wt.% NaCl.

From Figure 10, the OCP curves of the AlCrTiZrMo HEA film and the film annealed at 400 °C have similar changes with ΔOCP values of 0.453 and 0.461, respectively. During the rapid sliding, the two curves remain stable due to the dynamic balance between activation and passivation within the wear track [38,39]. Additionally, the two COFs are 0.13 and 0.09, respectively (Table 5). As shown in Figure 11a,b, many adhesions and furrows are found on the abrasion track of the AlCrTiZrMo HEA film and the film annealed for 400 °C.

The dense microstructure of the AlCrTiZrMo HEA film after annealing at 600 °C and the increased H and E improve the resistance to external damage, resulting in a reduction in ΔOCP, COF and wear rate. The ΔOCP, COF and wear rate of the AlCrTiZrMo HEA film annealed at 800 °C are minimized to 0.055, 0.04 and 1.34 × 10^−6^ mm^−3^·N^−1^·m^−1^, respectively, and no obvious wear characteristics appear in the abrasion track, which means that the film is not destroyed. The AlCrTiZrMo HEA film annealed at 800 °C still has a stable and dense micromorphology, as well as high H and H/E, which makes it significantly resist corrosive media and mechanical damage, showing excellent tribocorrosion resistance [40,41].

## 4. Conclusions

In general, this work prepared the AlCrTiZrMo HEA film with strong amorphization by the novel Co-FCVAD. The effects of thermal shock on the microstructure, mechanical properties and tribocorrosion properties of the films are analyzed. The slow diffusion effect of the AlCrTiZrMo HEA film and the amorphous phase in the structure make the diffusion of oxygen elements difficult. When the AlCrTiZrMo HEA film is annealed at 800 °C for 1 h in air, the dense ZrTiO_4_ composite oxide layer formed on the surface effectively prevents the oxidation from continuing. In addition, the H and E of the film reach the maximum values of 20.88 GPa and 220.2 GPa, respectively. The stable dense microstructure and excellent mechanical properties of the AlCrTiZrMo HEA film annealed at 800 °C for 1 h shows the best tribocorrosion resistance with the smallest ΔOCP, COF and wear rate values of 0.055, 0.04 and 1.34 × 10^−6^ mm^−3^·N^−1^·m^−1^, respectively.

## Figures and Tables

**Table 1:** Process parameters for AlCrTiZrMo HEA films.

Parameter	Value
Target	Ti_0.5_Mo_0.5_/Al/Cr/Zr
Cathode current (A)	130/110/110/110
Filter coil current (A)	2
Positive bias voltage (V)	24
Duty cycle (%)	100
Negative bias voltage (V)	−100
Base pressure (Pa)	3 × 10^−3^

**Table 2:** Crystal structures and contents of the constituent elements in the AlCrTiZrMo HEA film and the values of *δ*, ∆*H_mix_* and ∆*S*_conf_.

	Al	Cr	Ti	Zr	Mo
Content/at.%	15.82	22.86	19.49	24.74	17.09
Radius/Å	1.43	1.27	1.45	1.60	1.40
Structure	FCC	BCC	HCP	HCP	BCC
δ	0.077				
$∆H_{mix}$/kJ/mol	−18.40				
$∆S_{conf}$/J/(K·mol)	13.55				

**Table 3:** Chemical compositions of AlCrTiZrMo HEA film after annealing in air at different temperatures of RT, 400 °C, 600 °C and 800 °C.

Temperature (°C)	Composition (at.%)					
	Al	Cr	Ti	Zr	Mo	O
RT	15.82	22.86	19.49	24.74	17.09	--
400	15.71	22.56	18.34	24.37	17.01	2.01
600	14.64	21.05	17.95	23.95	16.89	5.52
800	11.74	18.78	13.08	21.67	14.23	20.50

**Table 4:** Gibbs free energy for oxides of different elements.

Element	ΔG^θ^_T_/kJ/mol
Al	−1120.48 + 0.21422T
Cr	−746.840 + 0.17029T
Ti	−943.490 + 0.17908T
Zr	−1096.210 + 0.18912T
Mo	−505.080 + 0.16862T

**Table 5:** Characterization results of tribocorrosion of AlCrTiZrMo HEA film annealed at different temperatures in 3.5wt.% NaCl solution.

Sample	ΔOCP(V)	COF	Wear Rate (10^−6^ mm^−3^·N^−1^·m^−1^)
As-deposited	0.453	0.13	7.12
400 °C	0.461	0.09	7.01
600 °C	0.393	0.05	6.22
800 °C	0.055	0.04	1.34

## Data Availability

Not applicable.
